# Exploration the role of social, cultural and environmental factors in tendency of female adolescents to smoking based on the qualitative content analysis

**DOI:** 10.1186/s12905-022-01617-0

**Published:** 2022-02-11

**Authors:** Alireza Jafari, Mehrsadat Mahdizadeh, Nooshin Peyman, Mahdi Gholian-Aval, Hadi Tehrani

**Affiliations:** 1 Department of Health Education and Health Promotion, School of Health, Social Development and Health Promotion Research Center, Gonabad University of Medical Sciences, Gonabad, Iran; 2 Social Determinants of Health Research Center, Mashhad University of Medical Sciences, Mashhad, Iran; 3 Department of Health Education and Health Promotion, School of Health, Mashhad University of Medical Sciences, Mashhad, Iran

**Keywords:** Tobacco, Cigarettes, Socio-cultural factors, Female adolescents, Tendency to smoke, Qualitative study

## Abstract

**Background:**

The aim of this study was to explain the socio-cultural and environmental factors of smoking tendency in female adolescents.

**Methods:**

This qualitative content analysis study was conducted among Iranian female adolescents in Mashhad, Iran. The data was collected through semi-structured interviews with 20 female smokers. The duration of each interview varies from 30 to 70 min. Data collection and management of data were done using MAXQADA software version 10.

**Results:**

In exploration the effective socio-cultural and environmental factors in the tendency of female adolescents to smoking, six subcategories of role modeling of friends, membership in groups, parenting patterns, family modeling, the predisposing community, and the negative impact of the media were extracted.

**Conclusions:**

The results obtained in this study indicated that there is a need to formulate policies and adopt environmental and social laws to reduce smoking. The results also showed the effective role of parents in improving personal skills, creating a stress-free environment in the family, and controlling adolescent behavior. Therefore, it is necessary to pay attention to all social and cultural aspects in order to make the smoking prevention programs.

## Background

According to the report of the World Health Organization (WHO), in 2018, 23.6% of adults aged 15 and over used tobacco globally, and 80% of them were current smokers [1]. Globally, 12.1% of young people aged 13 to 15 use tobacco, and 7.8% of women use tobacco. Approximately 6.5% of 13 to 15-year-old adolescents smoke, 4% of which are female adolescents [1]. According to the WHO report, the current smoking rate among people over 15 years old in Iran is 14%, of which 3.5% are girls [1].

There are several factors involved in adolescents’ tendency to smoke. One of the reasons for female smoking is that in the past century, tobacco companies have done heavy advertising for women smoking in high-income and low-income countries [2]. The results of a study showed that smoking by friends and family members are other reasons for the increase in smoking rates among men and women [3]. Also, peer pressure, smoking attractiveness, tobacco use by family members, curiosity, and lack of appropriate options to reduce stress, exposure of children to cigarettes, adult and sibling smoking status, advertising activities of tobacco companies, easy access to cigar cigarettes, media, low cost of cigarettes, perceptions of smoking, perceived approval from smokers friends for smoking, beliefs of being useful psychologically, early usage of cigarettes by children, beliefs of being useful physically, perceived discrimination, and fatalism are effective factors in starting and continuing smoking by women [4–9].

The results of a systematic study in Ethiopia showed that adolescents who are under pressure from their peers are more likely to smoke [10]. The results of a study in China also showed that adolescents who both parents smoke will be twice as likely to smoke in the future than those who have only one parent smoker [11]. Another factor that increases the risk of smoking among adolescents is the parents’ divorce, single-parent families, family environment, and psychological factors [11, 12]. The results of a study in Europe showed that girls usually start smoking at the age of 16 [13] and having a smoking friend was one of the most important factors in the onset of smoking by adolescents [14]. The results of a study showed that exposure to film smoking and perception of smoking risk by smokers are effective factors for adolescent smoking [15]. The results of a study in Sweden showed that factors such as low level of education of parents, poor family status, drinking of alcohol, low self-esteem, and positive attitude towards smoking are effective factors for cigarette smoking in adolescents [16].

Due to the extensive socio-cultural changes that have taken place in recent years in different societies as well as in Iran, the attitudes of Iranian girls and women towards smoking have changed and their tendency to smoke has increased, and smoking from an abnormal phenomenon is becoming a normal behavior in society. In Iran, few studies have been conducted on the reasons for the tendency of girls and women to smoke, and these studies have been done more quantitatively. Quantitative methods are not always appropriate for understanding meanings, beliefs, and experiences. Therefore, to discover the phenomena, it is necessary to perform a qualitative study that provides a broad and rich description of the phenomena [17, 18].

Recently, in the study of health issues, there has been a great deal of interest in considering a qualitative approach. The focus in this approach is on the interview and the action orientation of the participants. How people express themselves in conversation is an additional layer in understanding how people try to change behavior. Qualitative studies can provide insight into semantic structure [19–21]. Due to the increasing prevalence of smoking among female adolescents in Iran, this qualitative study was conducted on the smoking tendency of female adolescents to explore the social, cultural and environmental factors that influence smoking behavior.

## Methods

This qualitative content analysis study was conducted among Iranian female adolescents in Mashhad, Iran (A big city located eastern Iran) in 2020. The participants were selected from those who met the inclusion criteria. Inclusion criteria included of Iranian adolescents who lived in Mashhad, smoked cigarettes for at least 1 year, have signed a written consent form, and were willing to participate in the study. In this study, sampling was selected purposefully and continued until the data was saturated. The estimated sample size in qualitative research depends on several factors. The results of the study suggest that it is important to estimate the size of the sample to achieve data saturation. Researchers should consider many factors, such as the scope and nature of the research, the quality of the data received, and the useful information obtained from each respondent [22–25].

In this study, after 17 interviews, the data was saturated, because the codes were duplicated and no new concepts were extracted. Also, three other interviews were carried out to ensure data saturation. Purposive sampling was designed to reflect diversity within a population rather than statistical generalizability or representativeness. Participants were selected with maximum variation from smoking places, parks, coffee shops, etc. Also, in the continuation of the study, research samples were included in the study through the snowball sampling method.

Before the interview, the participants were explained the research objectives. They were assured that their information was confidential and would only be used for the research objectives. In this study, data were collected from 20 female adolescent smokers using semi-structured interviews and face-to-face methods. In this study, questions were asked such as what socio-cultural factors have contributed in your tendency to smoke? and what environmental factors have contributed in your tendency to smoking? At the end of the interview, participants were asked to explain anything that was not mentioned. The duration of each interview varies from 30 to 70 min, depending on the location, participant’s tolerance, and environmental factors. In addition, contact information has been provided to the participants, so if they want to provide more detailed information, they can contact the researcher at another time.

In this study, the four standards of Lincoln and Guba (credibility, confirmability, transferability, and dependability) were used for data robustness [26, 27]. Credibility was checked by continuously observing the information, reviewing the material typed by the participant’s transcripts of dialogues, holding regular meetings with the research team. In order to verify the confirmability of the data, some parts of the transcripts of dialogues, free codes, and sub-categories have been reviewed by multiple experts, and necessary corrections have been made. Evaluate the transferability of data through purposeful sampling and selection of different participants, as well as data-saturated interviews. In order to check the dependability, a complete research protocol was drafted during the research process, the data collection process was accurately recorded. Also, the coding accuracy and reliability were measured by the research team’s encoder.

Data analysis was performed using the thematic analysis proposed by Nowell et al. which consists of six phases of familiarizing yourself with your data (Phase 1), generating initial codes (Phase 2), searching for themes (Phase 3), reviewing themes (Phase 4), defining and naming themes (Phase 5), and producing the report (Phase 6)[28]. In phase one, researchers familiarized with the collected data and preliminary understanding of possible emerging patterns. Phase 2 consist of the identification of initial codes across the dataset. Subsequently in phase 3 and phase 4, researchers identified codes and categorized systematically into different emerging themes and subthemes. Finally, researchers assign names to the overarching themes based on the main aspects of the data that they represent. In this study MAXQADA software version 10 was used to manage and analyze the interviews (For extract open coding, integrating open coding, creating subcategories, and extracting quotation).

## Results

This study was conducted through semi-structured interviews with 20 female adolescent smokers. The mean and standard deviation of the participants was 16.30 (± 2.70).Among the participants, 35% (n=7) were single children, 20% (n=4) had one brother, and 10% (n=2) had 2 brothers. Also, 10% (n=10) had one sister, 5% (n=1) had two sisters, and 5% (n=1) had three sisters. In response to the question “socio-cultural and environmental factors affecting the tendency of female adolescents to smoke” finally one category and six subcategories of role modeling of friends, membership in groups, parenting patterns, family modeling, the predisposing community, and the negative impact of the media were extracted. More complete results can be seen in Table 1.

**Table 1:** The view of adolescent female smokers about socio-cultural and environmental factors influencing their tendency to smoke

Maine category	Sub-category	Free codes
Socio-cultural and environmental factors	Role modeling of friends	Role modeling of friends/peer groups The importance of the role of friends Having female smoker friends Imitating of friends Interact with smokers
	Membership in groups	Become famous in cyberspace Prove yourself to others Gaining social popularity Admission in groups Attract the attention of others The importance of a group of smoking friends Being under the influence of friends Stimulate and encourage friends
	Parenting patterns	Family problems Parental divorce Stressful family environment Father’s indifference to family Pattern of inappropriate role of mother Parents’ indifference to hookah use Lack of proper communication between parents and children Inappropriate of parents’ upbringing style Parents’ neglect of their child’s smoking
	Family modeling	Modeling of smoking parents Exposure to smoking in the family Consumption of cigarettes smoking and alcohol consumption by the fathers Hookah consumption by parents
	The predisposing community	The not prohibition of smoking in the community Reducing the age of smoking High demand for cigarettes The unfavorable economic situation of the society The unfavorable environment of society Lack of proper recreational facilities for discharge of excitement The higher the cost of other entertainment than smoking Cigarettes available everywhere Low price of cigarettes Normalization of women’s smoking in society Sell cigarettes to any age group
	The negative impact of the media	Encouraging the media to smoke Media representation of cigarettes instead of presenting facts The negative role of social media Normalization of smoking by celebrities Advertise smoking in movies to attract more audience and sale more cigarettes

## Main category: Socio-cultural and environmental factors

### First subcategory: Role modeling of friends

Participants reported that factors such as the role model of friends/peers, the importance of the role of friends, having female smoker friends, imitation of friends., and interact with smokers were effective in adolescents’ tendency to smoke.

One teenager said that *“… My friends that using a cigarette to say to me that smoking calms them down when they are upset. Then when I was upset, I decided to try smoking once, which has the effect of smoking”.* Another teenager said that *“… I became a smoker in the group of my friends, they taught me how to smoke and when I tried it, I liked cigarette smoking”.*

### Second subcategory

*Membership in groups*

In this context, participants consider factors such as become famous in cyberspace, proving themselves to others, gaining social popularity, acceptance in groups, getting attention others, being under the influence of friends, stimulate and encourage friends were effective in adolescents’ tendency to smoke.

One participant stated that *“… It was very important for me to be socially popular and to be approved by my friends, and that’s why I smoked”.* Another teenager explained the role of persuading friends to start smoking by saying *“… The first time I smoked, my friend encouraged me to smoke and I did”.*

### Third subcategory: parenting patterns

Participants reported that factors such as family problems, parental divorce, stressful family environment, inappropriate the role modeling of mothers, use of hookah by parents, lack of proper communication between parents and adolescent, inappropriate of parents’ upbringing style, and parents’ neglect of their child’s smoking were child smokers were effective in adolescents’ tendency to smoke.

One of the teenagers about the effect of parental divorce on the tendency to use cigarettes mentioned that*“… My parents’ divorce played a big role in my tendency to smoke. Sometimes, I thought that if my parents had not separated, I would not have met many of my friends and I would not have had the freedom that I have now”.*

One teenager about parents’ wrong upbringing style said that *“…My parents’ upbringing style was not the same with each other at all, for example, my mother said do not do this, but my father said there is no problem and we always took refuge in my father because he gave us more freedom and parents’ upbringing style has been effective in my smoking”.*

### Fourth Subcategory

***Family modeling***.

In this regard, the participants stated that factors such as role modeling of smoking parents, exposure to smoking in the family, consumption of cigarettes and alcohol by fathers, and hookah consumption by parents were effective in adolescents’ tendency to smoke.

In exploration the role modeling of parents, one of the teenagers said that *“… Well, we always saw my father smoking and always smoking in front of us, and it created a positive attitude towards smoking in me and it was effective in my tendency to smoke”.*

Another participant stated that *“… I have been using hookah since I was a child. Hookah is a normal thing in my family. My parents use hookah, I also use it, and they don’t prohibit me from doing it. Well, hookah smoking has been effective in encouraging me to smoke”.*

### Fifth subcategory: the predisposing community

In exploration the predisposing community in adolescents’ tendency to smoke, participants mention that factors such as the not prohibition of smoking in the community, the availability of cigarettes everywhere, the low price of cigarettes, the normalization of women smoking in society, the unfavorable economic situation of the society, lack of proper recreational facilities for discharge of excitement, the high cost of other entertainment compared to cigarettes, and sell cigarettes to any age group were effective in adolescents’ tendency to smoke.

One participant said that *“…Well, the availability of cigarettes everywhere has affected my consumption, and I probably wouldn’t have come a long way to buy a cigarette if it weren’t so easily available”.*

In exploration the lack of cheap entertainment, one of the teenagers stated that *“… we could not go to sports, go to tennis, go to billiards or places like this, or they are very expensive and cost more than smoking a cigarette. I think smoking was an easier and cheaper entertainment, and I think that influenced my tendency to smoke”.*

### Sixth subcategory: the negative impact of the media

In xploration the negative impact of the media, factors such as encouraging the media to smoke, media representation of smoking instead of presenting facts, the negative role of social media, normalization of smoking by celebrities, and advertise of smoking in films to attract more audience and sale more cigarettes were influential in attracting and the tendency of teenagers to smoke.

In exploration the negative role of social media and networks, one teenager said *“… We have always seen celebrities smoking on social media and telling myself why I should not smoke, and this encourages me to “Smoking was effective”.*

In exploration the pattern of the role of celebrities in the tendency to smoke, one of the teenagers said that *“…I watched the episodes of a movie, which the film actor was constantly smoking, and when I saw the smoking of this actor, I wanted to smoke immediately and watched these scenes affected my smoking”.*

## Discussion

The aim of this study was to explain the socio-cultural and environmental factors of smoking tendency in female adolescents by qualitative content analysis. Based on the results, six subcategories of role modeling of friends, membership in groups, parenting patterns, family modeling, the predisposing community, and the negative impact of the media were extracted.

### Role modeling of friends

In this study, adolescents stated that factors such as the importance of the role of friends, having female smoker friends, imitation of friends, and interacting with smokers were effective in their tendency to smoke. The results of the Owusu-Sarpong study showed that smoking of close friends is effective in adolescents starting to smoke [29]. The results of various studies have shown that smoking by close friends, belonging to a smoking group, pressure from friends/peer group were the influencing factors of adolescent smoking. Smoking-related behaviors and attitudes of significant others (especially friends) are among the most consistent predictors of adolescent smoking. Modeling of friends’ smoking behavior appears to dominate the process of adolescents’ smoking initiation [30]. So how adolescents chosen their friends and the influence of peer groups are effective factors in predicting the onset of smoking in adolescents [31]. Therefore, the family must have sufficient supervision over the children’s choice of friends.

### Membership in groups

In this study, participants reported that factors such as become famous in cyberspace, proving themselves to others, gaining social popularity, acceptance in groups, getting attention others, being under the influence of friends, stimulate and encourage friends were effective in their tendency to smoke. The results of the Passey study showed that young girls use cigarettes to achieve social popularity and as a way to prove their identity and membership in groups [32]. The results of a study showed that social factors such as peer groups pressure and acceptance among friends play an important role in increasing the tendency to smoke [33]. The results of a qualitative study by Karimi showed that from the perspective of teachers, parents, and students, the most important factor in smoking in adolescents smoking was the feeling of looking good and getting attention from others [34]. If adolescents are encouraged to smoke to join peer groups and be accepted by their friends, smoking rates may increase.

### Parenting patterns

In this study, adolescents reported that factors such as family problems, parental divorce, stressful family environment, inappropriate the role modeling of mothers, use of hookah by parents, lack of proper communication between parents and adolescent, inattention, and parents’ neglect of their child’s smoking were effective in their tendency to smoke. The results of the Owusu-Sarpong study showed that parental divorce is one of the effective factors in tendency of adolescents to smoke [29]. The results of another study on women showed that family factors, including whether there are smokers in the family, family problems, and family constraints were effective in adolescents’ tendency to smoke [33]. The results of a qualitative study showed that fights and conflicts at home, excessive freedom of the child, non-consultation of parents with adolescents, and divorce of parents are the most important factors in adolescents’ tendency to smoke [35]. Based on the results, adolescents with higher self-esteem and parental supervision on their children’s performance are protective factors in the onset of smoking by adolescents [36].

### Family modeling

In the present study, the participants stated that factors such as role modeling of smoking parents, exposure to smoking in the family, consumption of cigarettes and alcohol by fathers, and hookah consumption by parents were effective in adolescents’ tendency to smoke. The results of a study showed that the most important external factors affecting smoking by female students were smoking by parents and the presence of a smoker at home [37]. The results of another study showed that the risk of smoking among adolescents is significantly increased due to factors such as siblings smoking, mothers smoking, use of hookah by teenagers, and use of hookah among family members [38]. Family tobacco use is one of the predictors of early smoking among adolescents [39]. Parents as a role model for adolescents can be an important factor in adolescents’ tendency to smoke. Especially when the consumer parents are hero in the child’s dream, this role modeling will be doubled. Given that many adolescents imitate their parents’ behaviors, it is necessary for parents to avoid high-risk behaviors or not to perform these behaviors in front of their children.

### The predisposing community

Based on the results of this study, participants reported that factors such as the not prohibition of smoking in the community, the availability of cigarettes everywhere, the low price of cigarettes, the normalization of women smoking in society, the unfavorable economic situation of the society, lack of proper recreational facilities for discharge of excitement, the high cost of other entertainment compared to cigarettes, sell cigarettes in attractive stands, sell cigarettes to any age group were effective in adolescents’ tendency to smoke. The results of a qualitative study showed that the availability and cheap price of cigarettes are effective factors in adolescents’ tendency to smoke [35]. The results of another qualitative study showed that the lack of healthy entertainment and the not prohibition of smoking in public places are the most important reasons for the tendency to smoke [40]. As a result of following social fashion, a large proportion of girl adolescents have a tendency to smoke. This shows that whenever a behavior is popular in society, girls do not pay attention to its harm. For them, following the fashion in society is more important than maintaining their health.

### The negative impact of the media

Results of this study showed that factors such as encouraging the media to smoke, media representation of smoking instead of presenting facts, the negative role of social media, normalization of smoking by celebrities, smoking style of celebrities, and advertise of smoking in the films were effective in adolescents’ tendency to smoke. The results of the Passey study showed that four relevant factors for understanding the social context in which girls start smoking were included colonization and tobacco use, the normalization of smoking in social networks, poor and stressful lives, and the importance of maintaining relationships in extended families and social networks [32]. The results of a systematic review study by Wellman showed that exposure to movies showing smoking is effective for adolescent smoking [36]. Based on the results of a qualitative study, students considered an imitation of celebrities as the main socio-environmental factor of smoking [34]. Tobacco companies have done a lot of advertising on women smoking in recent years. These companies try to change the cultural meaning of smoking in the community and remove cultural barriers through various advertisements. With the removal of these barriers, the social pressures related to smoking by women have decreased and the rate of smoking has increased [2]. Exposure of adolescents to media with smoking content increases their tendency to smoke. The age of exposure to the media and the widespread use of daily media, as well as the exposure of young people without parental supervision, and the lack of media literacy skills can lead to the tendency of young people to smoke.

## Conclusions

The results obtained in this study indicate that there is a need for new policies and environmental and social laws for smoking reduction. The results also showed the effective role of parents in improving personal skills, creating a stress-free environment in the family, and controlling adolescent behavior. Therefore, to prevent the prevalence of smoking among young people, it is necessary to pay attention to all social and cultural aspects that are effective in this field, so that smoking prevention programs can be more effective.

## Data Availability

The data sets used and/or analyzed during the current study was available from the corresponding author on reasonable request.

## Abbreviations

WHO: World Health Organization.
